# Total Delay Is Associated with Unfavorable Treatment Outcome among Pulmonary Tuberculosis Patients in West Gojjam Zone, Northwest Ethiopia: A Prospective Cohort Study

**DOI:** 10.1371/journal.pone.0159579

**Published:** 2016-07-21

**Authors:** Senedu Bekele Gebreegziabher, Gunnar Aksel Bjune, Solomon Abebe Yimer

**Affiliations:** 1 Amhara Regional State Health Bureau, Bahir Dar, Ethiopia; 2 Department of Community Medicine, Institute of Health and Society, University of Oslo, Oslo, Norway; 3 Oslo University Hospital, Oslo, Norway; 4 Department of Bacteriology and Immunology, Division of Infectious Disease Control, Norwegian Institute of Public Health, Oslo, Norway; University of Malaya, MALAYSIA

## Abstract

**Background:**

delay in diagnosis and treatment of tuberculosis (TB) may worsen the disease, increase mortality and enhance transmission in the community. This study aimed at assessing the association between total delay and unfavorable treatment outcome among newly diagnosed pulmonary TB (PTB) patients.

**Methods:**

A prospective cohort study was conducted in West Gojjam Zone, Amhara Region of Ethiopia from October 2013 to May 2015. Newly diagnosed PTB patients who were ≥15 years of age were consecutively enrolled in the study from 30 randomly selected public health facilities. Total delay (the time period from onset of TB symptoms to first start of anti-TB treatment) was measured. Median total delay was calculated. Mixed effect logistics regression was used to analyze factors associated with unfavorable treatment outcome.

**Results:**

Seven hundred six patients were enrolled in the study. The median total delay was 60 days. Patients with total delay of > 60 days were more likely to have unfavorable TB treatment outcome than patients with total delay of ≤ 60 days (adjusted odds ratio [AOR], 2.33; 95% confidence interval [CI], 1.04–5.26). Human immunodeficiency virus (HIV) positive TB patients were 8.46 times more likely to experience unfavorable treatment outcome than HIV negative TB patients (AOR, 8.46; 95% CI, 3.14–22.79).

**Conclusions:**

Long total delay and TB/HIV coinfection were associated with unfavorable treatment outcome. Targeted interventions that can reduce delay in diagnosis and treatment of TB, and early comprehensive management of TB/HIV coinfection are needed to reduce increased risk of unfavorable treatment outcome.

## Introduction

Tuberculosis (TB) remains a major threat to human beings, with the majority of cases occurring in the developing world. According to a recent World Health Organization (WHO) report, there were 9.6 million new TB cases and 1.5 million deaths from TB worldwide [1]. The 22 high TB burden countries collectively accounted for 80% of all estimated incident cases.

Ethiopia is among the 22 high TB burden countries in the world. The directly observed treatment, short-course (DOTS) strategy has been adopted in the country since 1992 to control the TB epidemic [2]. The prevalence, incidence and mortality from TB in Ethiopia is currently estimated at 200/100,000 population, 207/100,000 population and 33 per 100,000 population, respectively [1]. These indicators show that the TB burden in Ethiopia is still enormous.

Early diagnosis and prompt initiation of treatment is essential for an effective TB control program. Delay in TB diagnosis and treatment plays a major role in increasing the size of the infectious pool of TB. It may worsen the disease, increase the risk of mortality and enhance transmission in the community [3]. Delay in diagnosis and treatment of TB has previously been studied in various parts of the world. A systematic review reported that the median time of delay from onset of cough until treatment initiation varied from 21–136 days [4]. In Ethiopia, a number of studies that assessed the length of time of delay in TB diagnosis and treatment showed long delay time from onset of symptoms till initiation of treatment [5–9]. Various factors such as rural residence [8], lower educational level [7,9], being women, large family size, and stigma [9], being old age [8], first visit to non-formal health providers [7], first visit to clinics/ health posts [6] and form of TB [6,8] were reported as predictors of total delay.

Despite the increasing number of studies on diagnostic and treatment delay however, there is little to no information about the effect of total delay on unfavorable treatment outcome in Ethiopia and elsewhere. Factors such as old age, gender, TB/HIV coinfection, rural residence, low educational level and retreatment were reported to be associated with unfavorable treatment outcome [10–14]. Investigating the consequences of total delay on TB treatment outcome is important to suggest interventions that will improve the treatment success rate. Thus, the aim of this study was to analyze the association of total delay with unfavorable treatment outcome.

## Methods

### Study Setting

This study was conducted in West Gojjam Zone which is one of the ten zones of Amhara Region, Ethiopia. The total population is estimated at 2 382 497 [15]. More than 90% of the population resides in rural areas. One government hospital, 90 government health centers, 356 health posts and 76 private health institutions were rendering health services to the population during the study period.

A health post is the lowest level health care and is staffed by two female health extension workers (HEWs). HEWs play an important role in identifying and referring TB suspects to the next level of health care i.e. health centers for TB diagnosis and initiation of treatment. Health posts are not equipped with TB diagnostic tools.

### TB Diagnosis and Treatment

The national guideline for clinical and programmatic management of TB which is adapted from the WHO TB treatment guidelines was followed to TB diagnosis, TB classification, case definition, TB treatment and evaluate treatment outcome [16]. Smear-positive TB is diagnosed when a patient has at least two initial sputum smear examinations positive for acid-fast bacilli (AFB), or one initial sputum smear examination positive for AFB and culture positive, or one initial sputum smear examination positive for AFB and radiographic abnormalities consistent with active TB. Smear-negative TB is diagnosed when a patient has symptoms suggestive of TB with at least three initial sputum smear examinations negative for AFB, radiographic abnormalities consistent with active TB, no response to a course of broad spectrum antibiotics and a decision by a clinician to treat with a full course of anti-TB chemotherapy. Provider initiated HIV counseling and testing is one of the routine clinical services given to TB patients.

TB treatment for new TB patients is given for six months. The regimen contains an intensive phase of daily chemotherapy with (2RHZE) rifampicin, isoniazid, pyrazinamide and ethambutol for two months, followed by a continuous phase treatment with (4RH) rifampicin and isoniazid for four months [16].

In this study, treatment outcomes are categorized into successful and unfavorable treatment outcomes. Successful treatment outcome includes “cured” and “treatment completed” cases. A patient is defined as “cured” if he/she became smear or culture negative in the last month of treatment and on at least one previous occasion. A patient is labeled as “treatment completed” if he/she completed treatment with resolution of symptoms [16, 17].

Unfavorable treatment outcome includes “treatment failure” cases and patients who “died”. A patient, who becomes smear or culture positive at five months or later during treatment or harbors a multi-drug resistant (MDR) strain at any point of time during the treatment, is referred to as “treatment failure”. A patient who died for any reason during treatment for TB is defined as “died”.

Defaulter: a defaulter is a patient who has been on treatment for at least four weeks and whose treatment was interrupted for eight or more consecutive weeks [16, 17].

### Study Design and Population

This is a prospective cohort study conducted from October 2013 to May 2015. All newly diagnosed PTB patients ≥ 15 years of age who were registered for treatment in the selected public health facilities of the study area were consecutively enrolled. Patients were prospectively followed throughout their treatment period (six months). Health workers working in TB clinics of each study site provided treatment, followed patients and recorded treatment outcomes. PTB patients below 15 years of age, extra pulmonary TB (EPTB) patients, and TB patients with a previous history of TB and MDR-TB cases were excluded from the study.

### Sample Size and Sampling Technique

The minimum required sample size for this study was determined using the formula for comparison of two proportions. This was done by applying openepi statistical software (version 2.3). The following assumptions were taken into account when calculating sample size. We considered 95% confidence interval, 80% power and exposed to non-exposed ratio of 1.0. The previous national report that showed 11% unsuccessful treatment outcome [18] was used to assign the proportion of exposed and non-exposed study participants with treatment outcome. Accordingly, we assumed that 8.5% of PTB patients exposed to long total delay and 2.5% of PTB patients non-exposed to long total delay would have unfavorable treatment outcome. Based on these assumptions, the sample size was calculated to be 518. We added 20% for non-responses and lost to follow-up cases and the total sample size was estimated at 622. However, we finally included all of the 706 new PTB patients that attended to the study sites during the study period.

Random sampling method was used to select study sites. First, we obtained list of all public health facilities providing TB diagnostic and treatment services in West Gojjam Zone. Accordingly, 73 health centers and one hospital were providing TB diagnostic and treatment services during the study period. Of these, 29 health centers were randomly selected. We also added one hospital which is the only available hospital in the study zone. This makes a total of 30 study sites.

### Data Collection

Socio-demographic and clinical data were collected using a pre-tested semi-structured questionnaire. The data among others included: age, sex, symptoms at presentation, knowledge on TB and time period from onset of symptoms to first start of treatment. Trained health officers and nurses at each study site collected the data. To assure quality of the data, frequent supervision was made by the principal investigator and other supervisors throughout the data collection period. The supervisors regularly checked the completeness, consistency and accuracy of the data.

### Data Analysis

Data were entered, cleaned and analyzed using Statistical Package for the Social Sciences (SPSS) IBM Version 22 (SPSS Inc. Chicago, IL, USA). The median total delay was used as a cut-off point to dichotomize the sample into delay and non-delay groups [5, 6, 9]. Descriptive statistics such as proportions and medians with interquartile ranges (IQRs) were computed. Mann-Whitney/Kruskal-Wallis tests were used to compare group differences in total delay. Categorical variables were compared using Fishers’ exact test and chi-square test. For each TB knowledge question a score of one was given for the correct answer and a zero score was given for incorrect responses. Then, total knowledge score and median were calculated. Finally, those with a total score of below the median value were classified as having poor knowledge whereas, those equal or above the median value were considered as having good knowledge.

Mixed-effect logistic regression model was used in order to adjust the clustering effects. Health facilities were held as random effect variable and other variables were used as fixed effects. Kaplan-Meier curve was used to estimate the probability of unfavorable treatment outcome of patients by delay status. Univariate and multivariate analysis were performed by using Stata statistical software V.14 (Stata Corporation, College Station, Texas, 77845 USA). Models were fit to analyze independent predictors of total delay, unfavorable treatment outcome and mortality. A two sided p-value of < 0.05 was considered statistically significant.

### Operational Definition of Variables

Total delay: the time period from the onset of TB symptoms to first start of anti-TB treatment.

Long total delay: if the time period from the onset of TB symptoms to first start of anti-TB treatment is more than the calculated median total delay period.

A new case of TB: is a patient who has never had treatment for TB or who had taken anti-TB drugs for less than one month.

### Ethical Approval

The Regional Committee for Medical Research Ethics (REK Øst) in Oslo, Norway and the National Research Ethics Review Committee (NRERC) in Addis Ababa, Ethiopia approved this study. In addition, letter of support and permissions to conduct the study in the local area were obtained from the local administrations. All participants were fully informed before written consents were taken. Then written informed consent was obtained from each participant who was willing to take part in this study. For those participants under the age of 18 years, written consent was obtained from their parents/legal guardians. The participants were assured about the confidentiality of the data.

## Results

### Characteristics of the Study Participants

A total of 706 newly diagnosed PTB patients were included in the study. Of these were 423 (59.9%) males and 283 (40.1%) females. The median age of the study participants was 30 years (IQR: 23–47 years). Of the total study participants, 61.6% were rural residents, 46.3% were farmers by occupation and 58.2% had no formal education (Table 1). Majority, 95% of the study participants reported to have taken less than two hours to reach at the nearest health facility (Table 2).

**Table 1:** Socio-demographic and clinical characteristics of the study participants, October 2013 to May 2015, West Gojjam Zone, Ethiopia.

Variables	Frequency	Percent (%)
**Age (years)**		
15–24	197	27.9
25–44	291	41.2
≥45	218	30.9
**Sex**		
Male	423	59.9
Female	283	40.1
**Place of residence**		
Urban	271	38.4
Rural	435	61.6
**Educational level**		
Not literate	411	58.2
Literate	295	41.8
**Marital status**		
Married	386	54.7
Single	205	29.0
Divorced	73	10.3
Widowed	42	5.9
**Occupation**		
Civil servant	41	5.8
Housewife	39	5.5
Student	76	10.8
Farmer	327	46.3
Day laborer	82	11.6
Merchant	47	6.7
Others	94	13.3
**Monthly family income (Birr)**^b^		
1–400	109	15.4
401–800	84	11.9
≥ 801	264	37.4
No regular income	249	35.3
**HIV sero-status**		
Positive	82	11.6
Negative	616	87.3
Not known	8	1.1
**Forms of TB**		
Smear-positive PTB	334	47.3
Smear -negative PTB	372	52.7

^**b**^ 1 USD = 22.00 Ethiopian Birr

PTB: Pulmonary tuberculosis

**Table 2:** Knowledge about TB and health seeking of the study participants, October 2013 to May 2015, West Gojjam Zone, Ethiopia.

Variables	Frequency	Percent (%)
**Knowledge of TB**		
Poor	355	50.3
Good	351	49.7
**Perceived to be stigmatized**		
Yes	88	12.5
No	618	87.5
**Time travelled to arrive at the nearest health facility** ^Ѱ^		
≤ 2 hours	671	95.0
> 2 hours	35	5.0
**Health facilities first visited**		
Private health facilities	280	39.6
Public health centers	342	48.4
Public hospitals	71	10.0
Health posts	13	2.0
**First action taken**		
Visited formal-health provider	433	61.3
Visited non-formal health provider	223	31.6
Self-treatment with home remedy	50	7.1

^**Ѱ**^ nearest health facility including health post, TB: tuberculosis

### Duration of Delay and TB Symptoms

**Fig 1:**
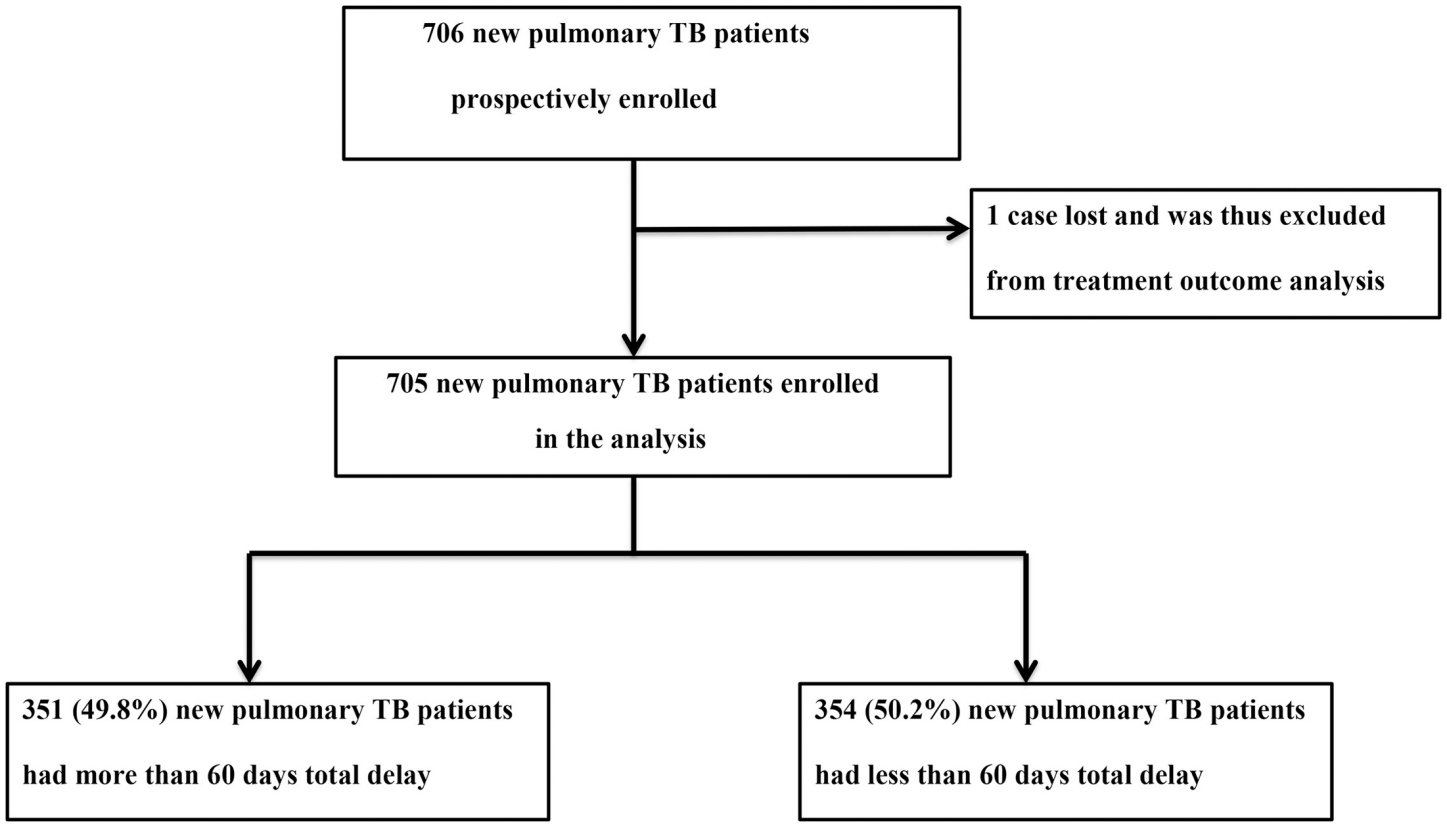
Study participants profile.

The median total delay was 60 days (IQR: 24–147 days). For 351 (49.8%) patients, the median total delay was > 60 days (Fig 1). Six hundred nineteen (97.9%) patients had persistent cough. High frequencies of fever, chest-pain, loss of appetite and body weight loss were reported among patients with total delay of > 60 days than those patients with total delay of ≤ 60 days (Table 3).

**Table 3:** Baseline signs and symptoms of new PTB patients stratified by median total delay, October 2013 to May 2015, West Gojjam Zone, Ethiopia.

Variables	Total number (%)	Total delay > 60 days	Total delay ≤ 60 days	P value
Cough	619 (97.9)	344 (97.7)	347 (98.0)	0.78
*Blood in sputum	167 (23.7)	94 (40.0)	73 (33.2)	0.13
Chest pain	578 (81.9)	311 (88.4)	267 (75.4)	< 0.001
Fever	513 (72.7)	296 (84.1)	217 (61.3)	< 0.001
Loss of appetite	633 (89.7)	334 (94.9)	299 (84.5)	< 0.001
Night sweats	569 (80.6)	285 (81.0)	284 (80.2)	0.80
Body weight loss	548 (77.6)	301 (85.5)	247 (69.8)	< 0.001

PTB: pulmonary tuberculosis

Note: Participants could have more than one symptom

*Calculated among patients who reported productive cough

### Factors Associated with Total Delay

In multivariate analysis, poor knowledge of TB (AOR, 2.39; 95% CI, 1.57–3.64), first visit to a non-formal health provider (AOR, 5.27; 95% CI, 3.43–8.08), being sputum smear-negative (AOR, 1.58; 95% CI, 1.09–2.29) and first visit to a health center (AOR, 2.18; 95% CI, 1.45–3.26) were factors associated with long total delay (Table 4).

**Table 4:** Associations of socio-demographic and clinical factors with total delay of new PTB patients, October 2013 to May 2015, West Gojjam Zone, Ethiopia.

Variables	Number	Total delay ^¶^ Delayed (%)	Crude OR (95% CI)	Adjusted OR (95% CI)
**Sex**				
Male	423	197 (46.6)	1.00	1.00
Female	283	155 (54.8)	1.46 (1.06–2.00)	1.32 (0.88–1.99)
**Age**				
15–24	197	86 (43.7)	1.00	1.00
25–44	291	136 (46.7)	1.13 (0.77–1.65)	0.86 (0.46–1.58)
≥45	218	130 (59.6)	2.03 (1.35–1.07)*	1.16 (0.57–2.35)
**Education**				
Literate	295	119 (40.3)	1.00	1.00
Not literate	411	233 (56.7)	2.17 (1.57–3.01)*	1.41 (0.84–2.38)
**Occupation**				
Civil servant	41	11 (26.8)	1.00	1.00
Housewife	39	16 (41.0)	1.91 (0.71–5.15)	0.41 (0.09–1.73)
Student	76	36 47.4)	2.58 (1.08–6.19)	1.25 (0.33–4.73)
Farmer	327	179 (54.7)	3.63 (1.68–7.86)*	1.29 (0.48–3.49)
Day laborer	82	36 (43.9)	2.22 (0.94–5.27)	0.65 (0.19–2.28)
Merchants	47	21 (44.7)	2.31 (0.89–6.01)	1.79 (0.57–5.64)
Others	94	53 (56.4)	3.91 (1.67–9.16)*	1.21 (0.34–4.33)
**Marital status**				
Married	386	195 (50.5)	1.00	1.00
Single	205	87 (42.4)	0.70 (0.49–1.01)	0.79 (0.44–1.44)
Divorced	73	44 (60.3)	1.44 (0.84–2.44)	1.22 (0.63–2.34)
Widowed	42	26 (61.9)	1.56 (0.79–3.10)	1.13 (0.46–2.75)
**Place of residence**				
Urban	271	121 (44.6)	1.00	1.00
Rural	435	231 (53.1)	1.48 (1.07–2.07)*	1.12 (0.71–1.78)
**Monthly family income (Birr)**^b^				
1–400	109	47 (43.1)	0.79 (0.49–1.27)	0.53 (0.22–1.23)
401–800	84	46 (54.8)	1.24 (0.74–2.09)	0.88 (0.34–2.26)
≥ 801	264	134 (50.8)	1.02 (0.70–1.48)	0.79 (0.32–1.94)
No regular income	249	125 (50.2)	1.00	1.00
**HIV sero-status**				
Positive	82	42 (51.2)	1.04 (0.64–1.70)	1.19 (0.64–2.26)
Negative	616	304 (49.4)	1.00	1.00
**Knowledge of TB**				
Poor	355	222 (62.5)	3.25 (2.32–4.54)*	2.39 (1.57–3.64)*
Good	351	130 (37.0)	1.00	
**Perceived to be stigmatized**				
Yes	88	42 (47.7)	0.92 (0.57–1.49)	0.84 (0.47–1.49)
No	618	310 (50.2)	1.00	1.00
**Distance to the nearest HF**				
≤ 2hrs	671	333 (49.6)	1.00	1.00
> 2hrs	35	19 (54.3)	1.45 (0.71–2.97)	0.73 (0.32–1.69)
**Forms of TB**				
Smear -positive PTB	334	150 (44.9)	1.00	1.00
Smear -negative PTB	372	202 (54.3)	1.55 (1.14–2.13)*	1.58 (1.09–2.29)*
**Health facilities first visited**				
Private health facilities	280	122 (43.6)	1.00	1.00
Public health centers	342	187 (54.7)	1.64 (1.17–2.29)*	2.18 (1.45–3.26)*
Public hospitals	71	36 (50.7)	1.25 (0.72–2.17)	1.26 (0.66–2.41)
Health posts	13	7 (53.8)	1.57 (0.49–4.99)	1.63 (0.41–6.53)
**First action taken**				
Visited formal-health provider	433	165 (38.1)	1.00	1.00
Visited non -formal health provider	223	161 (72.2)	5.12 (3.48–7.55)*	5.27 (3.43–8.08)*
Self-treatment with home remedy	50	26 (52.0)	1.98 (1.06–3.70)*	1.87 (0.93–3.76)

*P ≤ 0.05,

^¶^: > 60 days,

OR: Odds Ratio, CI: confidence interval, PTB: pulmonary tuberculosis HF: health facility,

^b^ 1 USD = 22.00 Ethiopian Birr

### Tuberculosis Treatment Outcome

Overall, 656 (93%) patients had a successful treatment outcome (cured and treatment completed). A total of 38 (5.4%) patients had unfavorable treatment outcome (treatment failure and death) (Table 5). The proportion of unfavorable treatment outcome was higher for patients with total delay of > 60 days (7.6%) than for patients with total delay of ≤ 60 days (3.4%) (P = 0.02).

**Table 5:** Treatment outcomes of the study participants, West Gojjam Zone, Ethiopia.

Treatment outcome	Number (%)
Cured	310 (44.0)
Treatment completed	346 (49.0)
Treatment failure	10 (1.4)
Died	28 (4.0)
Defaulter	11(1.6)

**Fig 2:**
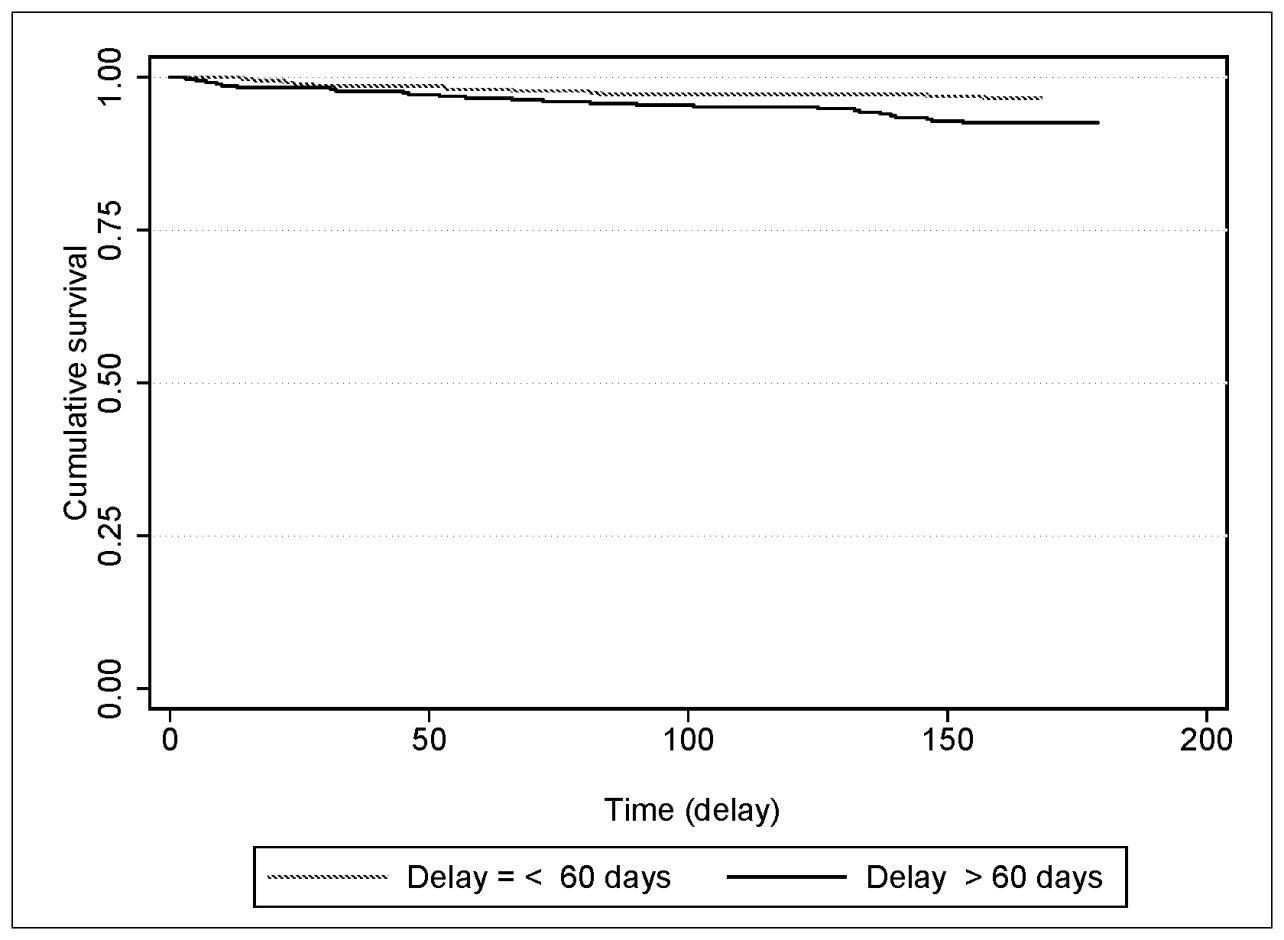
Kaplan-Meier estimate survival curves showing unfavorable treatment outcome of new PTB patients by total delay status.

In multivariate analysis, those patients with total delay of > 60 days were more likely to have unfavorable treatment outcome than those patients with total delay of ≤ 60 days (AOR, 2.33; 95% CI, 1.04–5.26) (Table 6) (Fig 2). Being HIV-positive was associated with unfavorable treatment outcome (AOR, 8.46; 95% CI, 3.14–22.79).

**Table 6:** Associations of socio-demographic and clinical factors with unfavorable treatment outcome of new PTB patients, October 2013 to May 2015, West Gojjam Zone, Ethiopia.

Variables	Number	Unfavorable treatment outcome (%)	Crude OR (95% CI)	Adjusted OR (95% CI)
**Total delay**				
≤ 60 days	354	12 (3.4)	1.00	1.00
> 60 days	351	26 (7.4)	2.25 (1.10–4.59)*	2.33 (1.04–5.26)*
**Sex**				
Male	423	26 (6.1)	1.00	1.00
Female	282	12 (4.2)	0.66 (0.33–1.36)	0.38 (0.14–1.01)
**Age**				
15–24	197	4 (2.0)	1.00	1.00
25–44	291	19 (6.5)	3.39 (1.12–10.22)*	2.14 (0.51–8.95)
≥45	217	15 (7.0)	3.69 (1.19–11.48)*	2.85 (0.61–13.40)
**Education**				
Literate	295	12 (4.1)	1.00	1.00
Not literate	410	26 (6.3)	1.65 (0.80–3.37)	1.24 (0.42–3.66)
**Marital status**				
Married	386	23 (6.0)	1.00	1.00
Single	205	7 (3.4)	0.56 (0.23–1.34)	1.94 (0.57–6.55)
Divorced/ Widowed	114	8 (7.0)	1.10 (0.47–2.59)	0.99 (0.34–2.91)
**HIV sero-status**				
Negative	615	22 (3.6)	1.00	1.00
Positive	82	15 (18.3)	6.61 (3.10–14.08)*	8.46 (3.14–22.79)*
**Knowledge of TB**				
Good	351	15 (4.3)	1.00	1.00
Poor	354	23 (6.5)	1.59 (0.79–3.15)	1.27 (0.53–3.08)
**Perceived to be stigmatized**				
No	617	38 (6.2)	1.00	1.00
Yes	88	0 (0.0)	Undefined	0 (0.00— —)
**Place of residence**				
Urban	271	17 (6.3)	1.00	1.00
Rural	434	21 (4.8)	0.79 (0.39–1.56)	0.94 (0.35–2.49)
**Occupation**				
Farmer	327	18 (5.5)	1.00	1.00
Housewife	39	5 (12.8)	2.30 (0.77–6.88)	1.90 (0.31–11.72)
Day laborer	81	6 (7.4)	1.32 (0.49–3.55)	0.37 (0.07–1.97)
Others	258	9 (3.5)	0.58 (0.25–1.34)	0.31 (0.08–1.24)
**Monthly family income (Birr)**^b^				
No regular income	248	16 (6.4)	1.00	1.00
1–400	109	6 (5.5)	0.85 (0.32–2.28)	0.61 (0.16–2.25)
401–800	84	5 (5.9)	0.94 (0.33–2.72)	0.60 (0.13–2.70)
≥ 801	264	11 (4.2)	0.65 (0.29–1.47)	0.45 (0.12–1.77)
**Forms of TB**				
Smear-positive PTB	333	18 (5.4)	1.00	1.00
Smear-negative PTB	372	20 (5.4)	1.04 (0.53–2.03)	0.75 (0.35–1.59)
**Distance to the nearest health facility**				
≤ 2hrs	670	37 (5.5)	1.00	1.00
> 2hrs	35	1 (2.8)	0.67 (0.08–5.27)	0.85 (0.09–7.99)

*P ≤ 0.05, OR: Odds ratio, CI: confidence interval, PTB: pulmonary tuberculosis,

^b^ 1 USD = 22.00 Ethiopian Birr

Among the 38 patients with unfavorable treatment outcomes, 28 (73.7%) died and 10 (26.3%) had treatment failure. Further analysis was performed to analyze the association between total delay and death of PTB patients. Of the 28 patients who died during TB treatment, 18 (64.3%) patients had a total delay of > 60 days, and 27 patients did know their HIV status of which 13 (48.1%) were coinfected with HIV. Of the 13 HIV coinfected PTB patients who died during treatment, 11(84.6%) had total delay of more than 60 days. Nineteen (67.8%) deaths occurred during the intensive phase treatment (the first two months). Of these, 12 (63.1%) patients had total delay of > 60 days. The multivariate analysis did not show statistically significant association between total delay and death during treatment (AOR, 1.62; 95% CI, 0.64–4.12) (Table 7). HIV-positive TB patients were 21.7 times more likely to die compared to HIV-negatives (AOR 21.74; 95% CI, 6.35–74.42).

**Table 7:** Factors associated with death of PTB patients, October 2013 to May 2015, West Gojjam Zone, Ethiopia.

Variables	Number	Death (%)	Crude OR (95% CI)	Adjusted OR (95% CI)
**Total delay**				
≤ 60 days	354	10 (2.8)	1.00	1.00
> 60 days	351	18 (5.1)	1.84 (0.83–4.08)	1.62 (0.64–4.12)
**Sex**				
Male	423	19 (4.5)	1.00	1.00
Female	282	9 (3.2)	0.69 (0.30–1.56)	0.35 (0.11–1.11)
**Age**				
15–24	197	3 (1.5)	1.00	1.00
25–44	291	12 (4.1)	2.80 (0.78–10.11)	1.33 (0.23–7.66)
≥45	217	13 (5.9)	4.18 (1.16–15.03)*	2.97 (0.47–18.82)
**Place of residence**				
Urban	271	12 (4.4)	1.00	1.00
Rural	434	16 (3.7)	0.84 (0.39–1.84)	1.00 (0.31–3.26)
**Occupation**				
Housewife	39	4 (10.3)	1.00	1.00
Farmer	327	14 (4.3)	0.39 (0.12–1.30)	0.45 (0.56–3.51)
Day laborer	81	3 (3.7)	0.33 (0.69–1.58)	0.05 (0.01–0.46)
Others	258	7 (2.7)	0.24 (0.66–0.89)	0.11 (0.18–0.69)
**Education**				
Literate	295	9 (3.0)	1.00	1.00
Not literate	410	19 (4.6)	1.56 (0.69–3.53)	1.53 (0.41–5.63)
**Marital status**				
Married	386	17 (4.4)	1.00	1.00
Single	205	5 (2.4)	0.54 (0.19–1.51)	1.94 (0.57–6.55)
Divorced/ Widowed	114	6 (5.3)	1.16 (0.44–3.06)	0.99 (0.34–2.91)
**HIV sero-status**				
Positive	82	13 (15.9)	8.52 (3.71–19.57)*	21.74 (6.35–74.42)*
Negative	615	14 (2.3)	1.00	1.00
**Monthly family income (Birr)**^b^				
No regular income	248	11 (4.4)	1.00	1.00
1–400	109	5 (4.6)	1.01 (0.34–3.03)	0.74 (0.16–3.44)
401–800	84	4 (4.8)	1.07 (0.33–3.51)	0.63 (0.10–3.80)
≥ 801	264	8 (3.0)	0.68 (0.26–1.73)	0.42 (0.85–2.13)
**Knowledge of TB**				
Good	351	11 (3.1)	1.00	1.00
Poor	354	17 (4.8)	1.56 (0.71–3.41)	1.27 (0.46–3.50)
**Forms of TB**				
Smear-positive PTB	333	8 (2.4)	1.00	
Smear-negative PTB	372	20 (5.4)	2.43 (1.04–5.66)*	2.24 (0.85–5.92)
**Distance to the nearest health facility**				
≤ 2hrs	670	27 (4.0)	1.00	1.00
˃ 2hrs	35	1 (2.9)	0.79 (0.09–6.26)	0.81 (0.85–7.53)

*P ≤ 0.05, OR: Odds ratio, PTB: pulmonary tuberculosis, CI: confidence interval,

^b^ 1 USD = 22.00 Ethiopian Birr

## Discussion

In this study, we found an association between total delay and unfavorable treatment outcome. Patients with total delay of > 60 days were found to be more likely to have unfavorable treatment outcome than patients with total delay of ≤ 60 days. Long delays in diagnosis and treatment start contribute to severity and complications of illness that may result in poor treatment outcomes [19, 20]. It also increases the risk of developing anti-TB drug resistance leading to increased mortality rate, treatment failure and transmission of drug resistant TB strains in the community [21, 22]. This finding underscores the importance of early diagnosis and treatment of TB.

Majority, 18 (64.3%) patients who died during TB treatment had total delay of > 60 days. However, our analysis did not show statistically significant association between long total delay and increased death. This is consistent with the findings of earlier studies conducted in Gunea [23] and Vietnam [20]. In contrast, associations between diagnostic delay and increased mortality were reported from studies in China [24] and Taiwan [25]. These differences in findings among the studies may be related to variations in sample sizes, study populations, study settings and socio-economic backgrounds considered in the respective studies. The lack of significant association between total delay and death in our study may also be linked to the small number of observed deaths included in the sample analysis. Therefore, larger prospective cohort study is warranted to further investigate the effect of total delay on TB mortality.

Early mortality reflects advanced disease and could be attributed to delayed treatment and late diagnosis [26]. In our study, 67.9% of the deaths occurred during the two month (intensive phase) TB treatment period. Of these, 63.2% of patients had total delay of > 60 days. This finding suggests that expediting early diagnosis and treatment may reduce TB mortality.

The proportion of HIV positive TB patients accounted for 12% of the study population. This is somewhat higher than the recent 9.3% HIV prevalence observed in TB patients in Ethiopia [1]. There are variations in HIV prevalence among the different regions of Ethiopia. The Amhara Region where the study zone is located is one of the regions that have the highest HIV prevalence [27].

We found high risk of unfavorable treatment outcomes among HIV coinfected PTB patients. This is in line with findings of former studies [12–14]. Also, being HIV-positive was significantly associated with increased death of TB patients during treatment. This is consistent with the results of previous studies conducted in Ethiopia [28, 29], Cameroon [30], and Uzbekistan [31]. A number of possible explanations including severity of TB due to immune suppression, atypical clinical manifestation of TB, delay in diagnosis and treatment initiation have previously been reported for increased unfavorable treatment outcomes [32]. Immunological studies have also shown that host responses to *M*. *tuberculosis* enhance HIV replication which accelerates the natural progression of HIV and further depression of cellular immunity [33]. In addition decreased gut absorption of anti-tuberculous drugs leads to impaired treatment outcomes including death among TB/HIV coinfected patients [34].

In our study, about 80.4% of HIV coinfected PTB patients who died during treatment had total delay of > 60 days. This finding suggests that early comprehensive management of TB and HIV coinfection is required to reduce the risk of increased mortality and improve treatment outcome [35]. Early Antiretroviral treatment initiation improves survival in HIV-positive TB patients [36]. It also enables linkage between HIV and TB treatment programmes and could improve adherence [37]. Moreover, initiation of early antiretroviral treatment decreases incidence of HIV disease progression and has good tolerability [38].

The observed median total delay in our study is lower than that reported from an earlier study conducted in a comparable setting in the Amhara Region of Ethiopia [5], but higher than the study findings reported from other regions of Ethiopia [7,9,19]. It is also higher compared to the studies done in Nepal [21], Angola [39], Zimbabwe [40] and South Africa [41].

Various factors may have contributed to the relatively shorter total delay observed in our study compared to the former study that was conducted in a similar settings in the study region [5]. Improved access to diagnostic and treatment services, and the contribution of health extention workers in identifying and referring suspected TB cases to health facilities (health centers) for smear microscopy may be considered as the possible factors for the observed reduction in total delay. However, given the increased access to TB diagnostic and treatment services, and the integration of TB screening activities with the general health service in health care facilities of the study area, the observed median total delay in the current study is still very long.

In a recent qualitative study, a number of challenges that may have implications for long delay in TB diagnosis and treatment were reported from the study area. Among others, frequent interruptions of laboratory reagents and anti-TB drugs supplies, lack of diagnostic tools for smear-negative TB and lack of laboratory personnel in some health centers were identified [13]. Interventions directed at solving these challenges are essential to reduce long total delay observed in the study.

Majority of the TB patients at presentation had cough however, significantly higher proportion of clinical symptoms such as chest-pain, fever, loss of appetite and body weight loss were observed among patients with total delay of > 60 days than their counterparts. This is not surprising as prolonged delay results in disease progression and worsening of symptoms. The association between delay in TB treatment and clinical severity was reported from a study conducted in Gunea-Bissau [23].

Fifty four percent of patients with total delay of more than 60 days first visited public health centers. This finding is similar with a previous study done in Ethiopia [8]. Smear microscopy is the only TB diagnostic tool used in majority of health centers of the study area. Smear microscopy has very low sensitivity [42] and may show false negative results in many TB patients. The study highlights the need to enhance TB diagnostic capacity of health facilities particularly health centers. Better TB diagnostic tools to complement AFB smear microscopy are needed to early diagnose PTB cases.

Our study has limitations. The study did not include extra pulmonary and retreatment TB cases. In addition, the study was only carried out in government health facilities. Therefore, the findings cannot be generalized to all TB patients in the study area. Patients may not exactly remember the onset of their symptoms and time of first vist to a medical provider. This may be subjected to a recall bias. However, efforts were made to lessen recall bias by using local calendars listing main religious and national days to define the perceived date of onset cough (TB symptoms) and time of first health seeking. We did not collect data about the level of treatment adherence of each patient. In addition, complete sputum smear grading data was lacking due to absence of mycobacterial culture facilities in the study zone. The non-inclusion of these two variables in the analysis may have an effect on our findings.

The study also has strengths. As far as our literature review is concerned, this is the first study in Ethiopia that attempted to investigate the effect of total delay on TB treatment outcome. Therefore, the findings may be used as baseline data for future studies. The study also covered a large geographic area and included patients from urban and rural settings. In addition, the study was prospective cohort study. Participants were properly followed until completion of their treatment and the proportion of lost to follow-up cases was very low.

## Conclusions

Long total delay and TB/HIV coinfection were associated with unfavorable treatment outcome. Mortality among HIV infected TB patients was high. Targeted interventions that can reduce delay in diagnosis and treatment of TB are needed to improve treatment outcome. Better TB diagnostic tools to complement AFB smear microscopy are needed at health centers of the study area. In addition, early comprehensive management of TB and HIV coinfection is essential to reduce the risk of increased mortality among HIV infected TB patients.
